# Cathepsin L secretion by host and neoplastic cells potentiates invasion

**DOI:** 10.18632/oncotarget.27182

**Published:** 2019-09-17

**Authors:** Samantha S. Dykes, Henrietta O. Fasanya, Dietmar W. Siemann

**Affiliations:** ^1^Department of Radiation Oncology, University of Florida, Gainesville, FL, USA

**Keywords:** breast cancer, macrophage, invasion, cathepsin L

## Abstract

The presence of macrophages within breast tumors correlates with metastatic potential. These tumor-associated macrophages often take on a pro-tumorigenic (M2-like) phenotype resulting in the secretion of growth factors and proteases, including the lysosomal protease cathepsin L. Since cathepsin L also is frequently secreted by breast cancer cells and contributes to tumor invasion, metastasis, and angiogenesis, we hypothesized that secretion of cathepsin L by both tumor-associated macrophages and neoplastic cells would facilitate the metastatic phenotype. Our results showed that the novel cathepsin L/K inhibitors KGP94 and KGP207 could inhibit *in vitro* M2 macrophage invasion and reduce the macrophage-stimulated invasion of 4T1 murine breast cancer cells. KGP94 and KGP207 treatment also reduced the expression of several M2-associated markers, suggesting that cathepsin L activity may be important for IL-4-driven M0 to M2 differentiation. In addition, cathepsin L shRNA knockdown studies revealed that cathepsin L from both the tumor cell and the macrophage population is important for tumor cell invasion. Thus our data suggest that tumor cells and macrophages may both contribute to the cathepsin L-driven metastatic phenotype of breast cancer. Taken together, these studies highlight the importance of cathepsin L in macrophage functions and suggest that cathepsin inhibition strategies may be therapeutically beneficial by impairing the progression of tumors with high infiltration of M2 macrophages.

## INTRODUCTION

Breast cancer is the most frequently diagnosed cancer and is the second leading cause of cancer-associated deaths in American females [1]. It is estimated that over 40,000 breast cancer deaths occur annually in the United States alone [1]. As with most solid tumors, breast cancer deaths are typically the consequence of metastases, rather than from failure to control the primary tumor. Therefore, it is essential to identify the mechanisms that contribute to the metastatic phenotype of breast cancer.

Proteases are catalytic enzymes that are often overexpressed in invasive tumors [2, 3]. There are several families of proteases that are associated with cancer, including, but not limited to, matrix metalloproteases (MMPs) and cathepsins. Proteases facilitate many aspects of the metastatic cascade including 1) integrin turnover during motility and invasion, 2) degradation of the extracellular matrix and basement membrane, 3) matrix-sequestered growth factor release, and 4) angiogenesis initiation [2, 3]. Both the MMP and cathepsin families of proteases contain proteases with overlapping substrates and functions [4, 5]. Due to the strong correlation between proteolytic activity and tumor grade, the development of protease inhibitors for use as anti-cancer drugs has been actively pursued. Initial studies focused on MMPs, but the lack of specificity and resulting side effect profile of these MMP inhibitors contributed to their failure in clinical trials [6, 7].

The cathepsins, most notably cathepsins B and L, also have been associated with aggressive tumor behavior and heightened invasive capacity of neoplastic cells [8, 9]. For example, cathepsin L is often overexpressed in invasive breast cancer cells and correlates with metastatic disease and poor prognosis [10, 11]. Cathepsin L is a ubiquitously expressed cysteine protease that is localized within the lysosome under normal conditions. However, cancer cells secrete cathepsin L to aid in invasion, metastasis, and initiation of angiogenesis [12]. Although there currently are no cathepsin inhibitors clinically available, several agents are under active development including KGP94 (3-bromophenyl-3-hydroxyphenyl-ketone thiosemicarbazone), a selective inhibitor of cathepsins L and K [13–16]. Indeed, our previous studies revealed that KGP94 reduces invasion of prostate and breast cancer cells *in vitro* and tumor-induced angiogenesis and prostate bone metastases *in vivo* [17, 18]. A second cathepsin L and K inhibitor, KGP207, differs structurally from KGP94 (an extra carbonyl group and phenyl ring) and does not bear the same functionalization pattern as KGP94 [13–16]. Both KGP94 and KGP207 demonstrate activity in the nM range.

Another key feature of aggressive breast cancers is the presence of macrophages. Macrophages play a significant role in the maintenance of normal breast tissue and in breast carcinoma [19, 20]. Their presence within the primary tumor correlates with disease progression and metastatic incidence [19, 21–23]. While classically studied for their role as pro-inflammatory phagocytes, macrophages can take on different characteristics in response to various cytokine stimuli. For example, un-stimulated macrophages (M0) can take on an anti-inflammatory (M2) role in response to IL-4 (IL-4) and interleukin-13 during wound healing and carcinogenesis [24–26]. The M2 stimulated macrophages contribute to multiple aspects of the metastatic cascade, including extracellular matrix remodeling leading to tumor cell invasion, promoting angiogenesis, and facilitating tumor cell entry into the vasculature [27–29]. Due to their contribution to multiple aspects of tumor progression, M2 macrophages may represent an attractive target for antitumor therapy [30]. One hallmark of M0 to M2 differentiation is the increased expression of multiple proteases, including cathepsin L [31–34].

We hypothesized that secretion of the proteolytic enzyme cathepsin L by both tumor-associated macrophages and neoplastic cells facilitates tumor cell invasion, a key element of metastasis. Our data indicate that cathepsin L inhibition using KGP94 or KGP207 significantly reduces the invasive potential of both tumor cells and macrophages. Furthermore, genetic knockdown of cathepsin L in either tumor cells or macrophages reduces tumor cell invasion in Boyden chambers. Interestingly, cathepsin L inhibition in macrophages may be altering macrophage M0 to M2 differentiation. Overall, these data suggest that cathepsin L is a potential target to prevent macrophage-driven breast cancer invasion.

## RESULTS

### Interleukin-4 stimulates cathepsin L expression in macrophages

**Figure 1:**
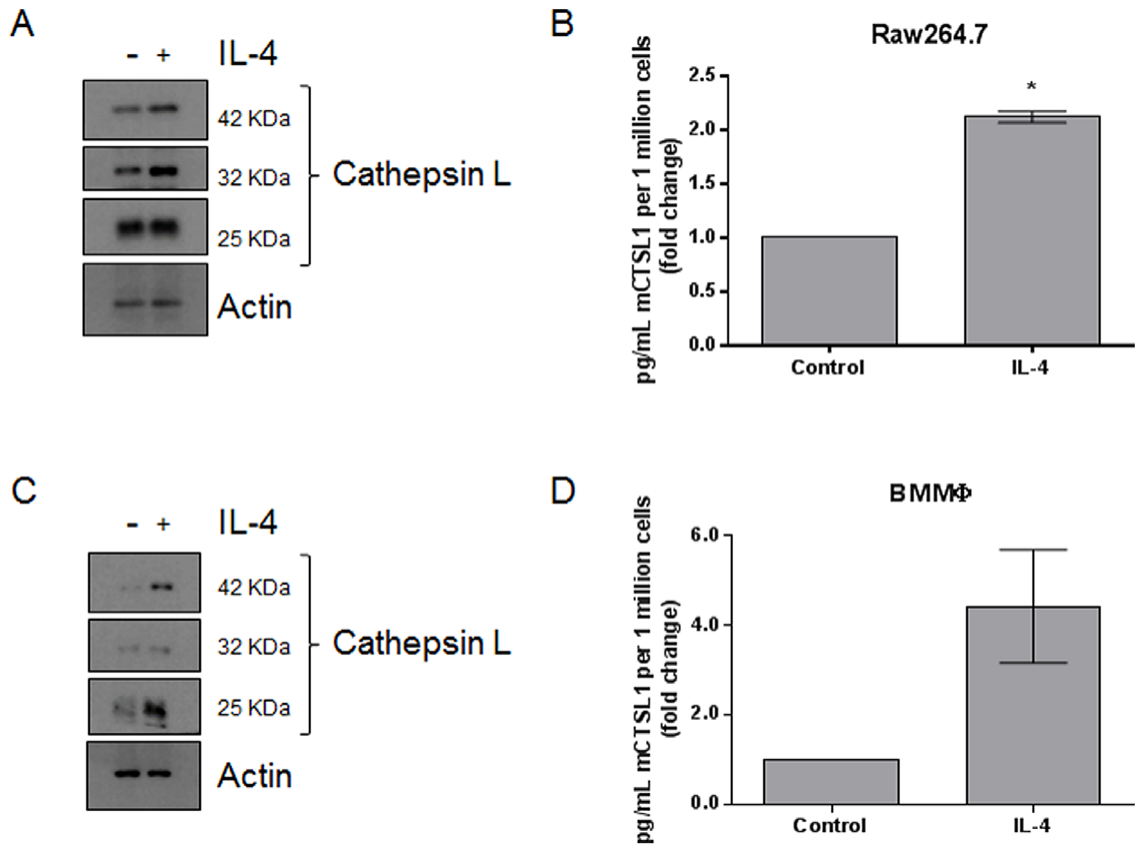
IL-4 upregulates the expression and secretion of cathepsin L from macrophages **(A)** Raw264.7 and **(C)** bone marrow-derived macrophages were stimulated with 10 ng/mL IL-4 for 48 h. Whole cell lysates were collected and analyzed by immunoblot. **(B)** Raw264.7 and **(D)** bone marrow-derived macrophages were stimulated with 10 ng/mL IL-4 for 48 h. Conditioned media was collected and analyzed by ELISA. ^*^=P <0.05

Previous studies have found that macrophages upregulate the expression of lysosomal proteases in response to IL-4 stimulation [31–34]. We treated Raw264.7 macrophages and primary bone marrow derived macrophages with 10 ng/mL IL-4, respectively. Semi-quantitative PCR indicated that IL-4 treatment resulted in the upregulation of M2-associated transcripts, including MRC-1, IL-10, and Fizz1, suggesting that IL-4 is causing an M0 to M2 transition (Supplementary Figure 1). Whole cell lysates were analyzed by immunoblot and revealed that cathepsin L protein levels were upregulated in response to IL-4 (Figure 1A and 1C; quantified in Supplementary Figure 2A, 2B). Conditioned medium was also collected and cathepsin L levels were analyzed by ELISA. We found that cathepsin L was secreted from both Raw264.7 (Figure 1B) and primary bone marrow derived macrophages (Figure 1D) in response to IL-4. These data are in line with previous findings and suggest that M2-like macrophages produce and secrete more cathepsin L compared to unstimulated M0 macrophages.

### Cathepsin L is important for both macrophage and neoplastic cell invasion

**Figure 2:**
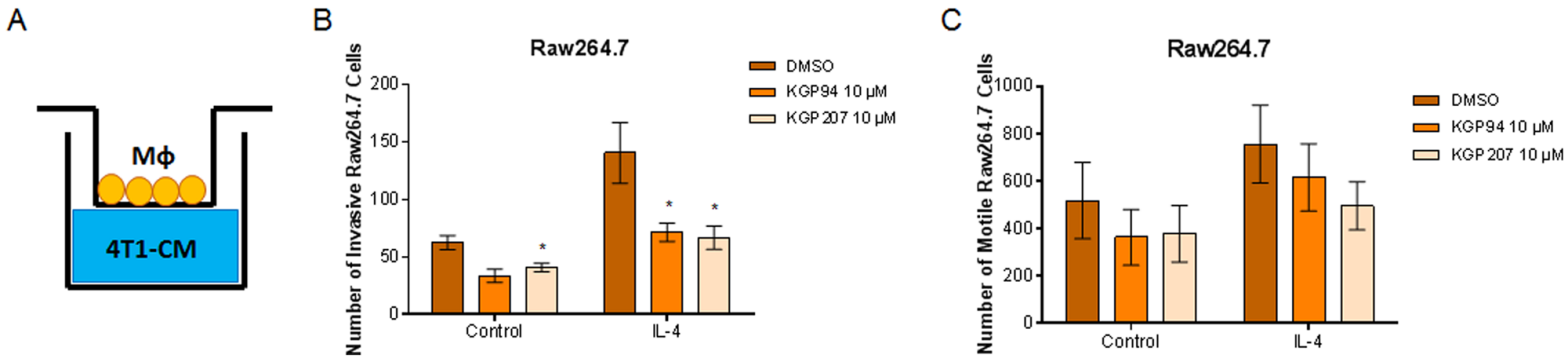
KGP94 and KGP207 reduce macrophage invasion **(A)** Experimental design. 4T1 conditioned-media was plated on the bottom of transwell inserts. Macrophages were treated in the presence or absence of 10 ng/mL IL-4 for 48 h prior to the start of transwell experiments. Control or IL-4 treated Raw264.7 cells were treated with KGP94 or KGP207 and allowed to **(B)** invade through Matrigel or **(C)** migrate toward 4T1 conditioned-media. ^*^=p <0.05 compared to DMSO.

**Figure 3:**
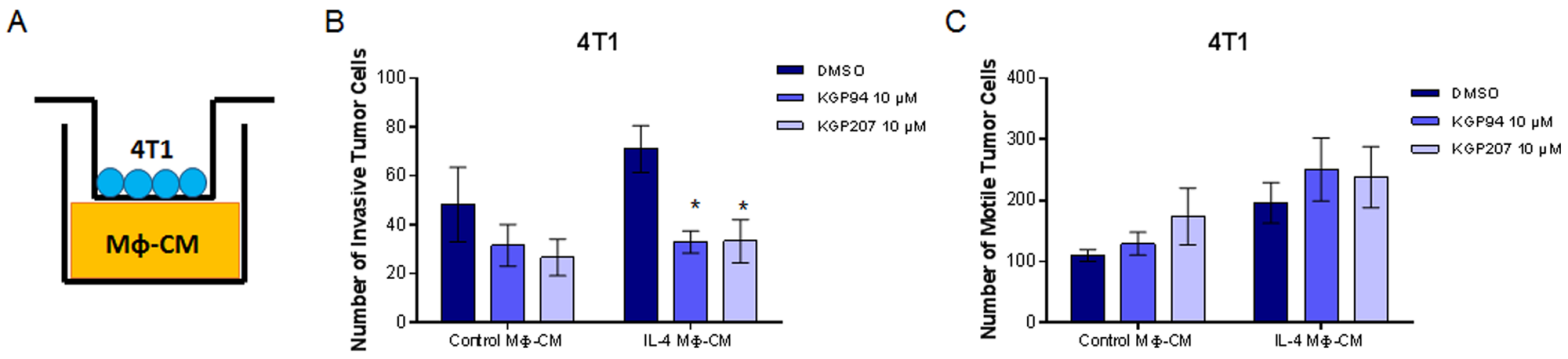
KGP94 and KGP207 reduce neoplastic cell invasion **(A)** Experimental design. Conditioned media from Raw264.7 cells treated with control or IL-4 was plated on the bottom of Boyden chambers. 4T1 tumor cells were treated with KGP94 or KGP207 and allowed to **(B)** invade through Matrigel **(C)** or migrate through Boyden chambers toward Raw264.7-conditioned media. ^*^=p <0.05 compared to DMSO.

Secreted proteases, including cathepsin L, are known to play a role in cell motility and invasion [35]. Our laboratory has previously shown that cathepsin L inhibition with KGP94 reduces invasion of breast and prostate cancer cells [17, 18, 36]. However, it is not known whether cathepsin L inhibition could reduce macrophage invasion. Using Boyden chambers, we tested whether inhibition of cathepsin L using KGP94 or KGP207 would alter the motility and invasiveness of macrophages (Figure 2A). Raw264.7 macrophages were stimulated with IL-4 for 48 hours prior to the start of the Boyden chamber assays. Macrophages were then treated with vehicle, KGP94, or KGP207 and allowed to invade through Matrigel (Figure 2B) or migrate in the absence of Matrigel (Figure 2C) toward 4T1 cell conditioned-media for 24 hours. We found that cathepsin L inhibition reduced invasion through Matrigel, but not motility of IL-4 stimulated macrophages. Similarly, 4T1 tumor cells were plated in the top chamber of Boyden chambers (Figure 3A) and allowed to invade through Matrigel (Figure 3B) or migrate in the absence of Matrigel (Figure 3C) toward macrophage-conditioned media for 24 hours. Cathepsin L inhibition with KGP94 or KGP207 reduced invasiveness, but not motility, of 4T1 cells. Together, these data indicate that cathepsin L activity is important for the invasive properties of both neoplastic cells and macrophages.

### Cathepsin L derived from both tumor cell and macrophage populations is important for tumor cell invasion

**Figure 4:**
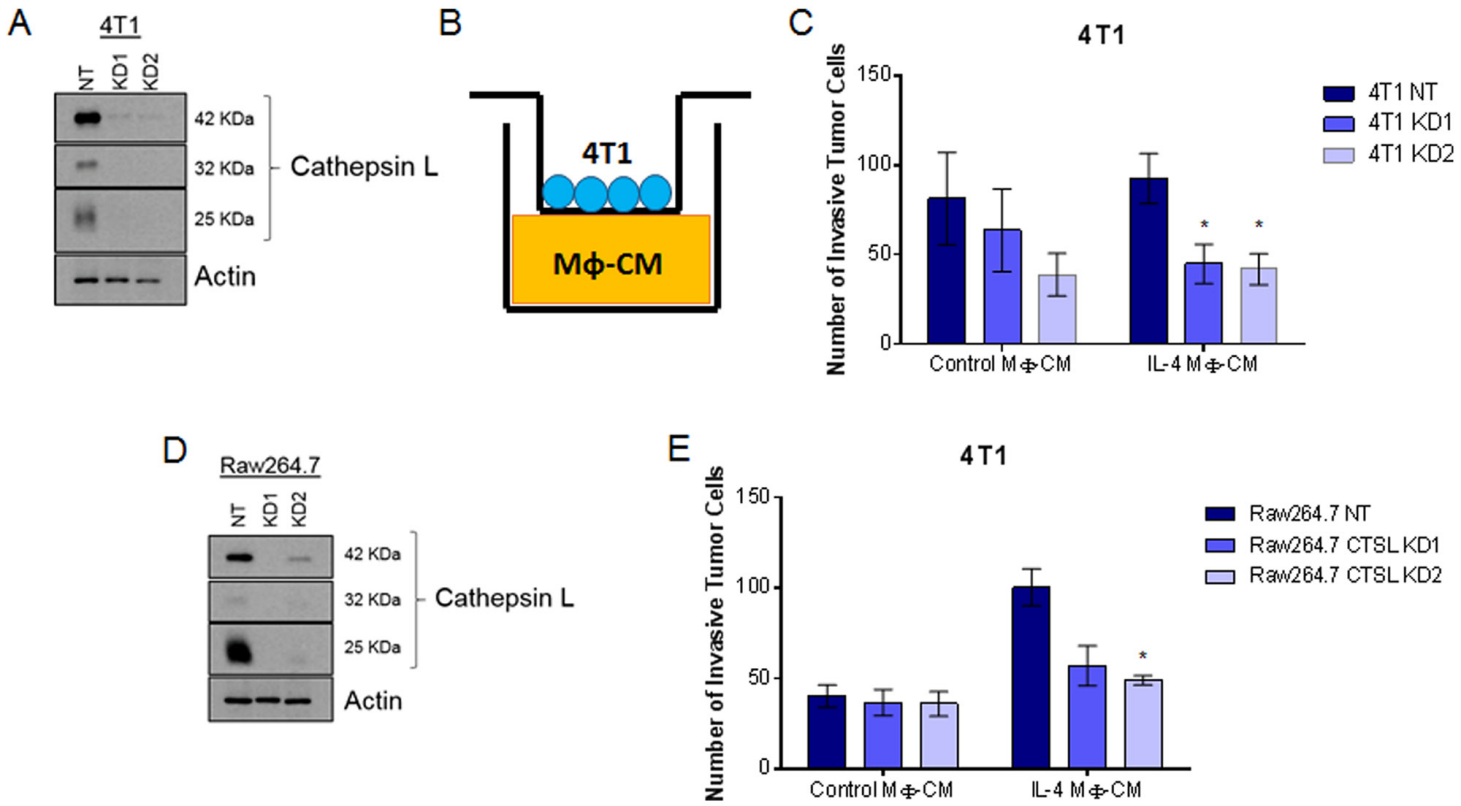
Cathepsin L knockdown in either the tumor cell or macrophage population reduces neoplastic cell invasion **(A)** Immunoblot of 4T1 cathepsin L knockdown. **(B)** Experimental design. Conditioned media from Raw264.7 cells treated with control or IL-4 was plated on the bottom of Boyden chambers. **(C)** 4T1 NT or cathepsin L KD tumor cells invaded through Matrigel toward Raw264.7 conditioned-media. **(D)** Immunoblot of Raw264.7 cathepsin L knockdown. **(E)** NT or CTSL KD macrophages conditioned-medium was harvested and plated on the bottom of transwell inserts. 4T1 tumor cells invaded through Matrigel toward NT or CTSL KD Raw264.7 conditioned-media. ^*^=p <0.05 compared to NT.

Cathepsin L is produced and secreted by both tumor cell and macrophage populations [9, 32]. However, it is not known which cell population produces the cathepsin L that is important for macrophage-stimulated tumor cell invasion. To test this, we utilized lentiviral-delivered shRNA to generate cathepsin L knockdowns in 4T1 breast cancer cells (Figure 4A; quantified in Supplementary Figure 2C). Non Target (NT) or cathepsin L knockdown (KD) 4T1 cells were then plated on the top chamber of Boyden chambers and allowed to invade through Matrigel toward macrophage-conditioned media (Figure 4B, 4C). Consistent with previously published results, cathepsin L knockdown in tumor cells reduced tumor cell invasiveness [17, 18, 36]. In order to test the contribution of macrophage-derived cathepsin L in tumor cell invasion we generated Raw264.7 cathepsin L knockdown cells (Figure 4D, quantified in Supplementary Figure 2D). Either NT or cathepsin L KD macrophages were stimulated with or without IL-4 for 48 hours and macrophage conditioned-media was plated on the bottom of the Boyden chamber inserts. 4T1 cells were plated on top of the inserts and allowed to invade through Matrigel toward NT or cathepsin L KD macrophage conditioned-media (Figure 4B, 4E). The results showed that depletion of cathepsin L in the macrophage population reduced the invasiveness of 4T1 cells. These data suggest that cathepsin L secreted by both tumor cells and macrophages contributes to tumor cell invasion.

### Cathepsin L activity plays a role in the expression of M2-associated proteins

**Figure 5:**
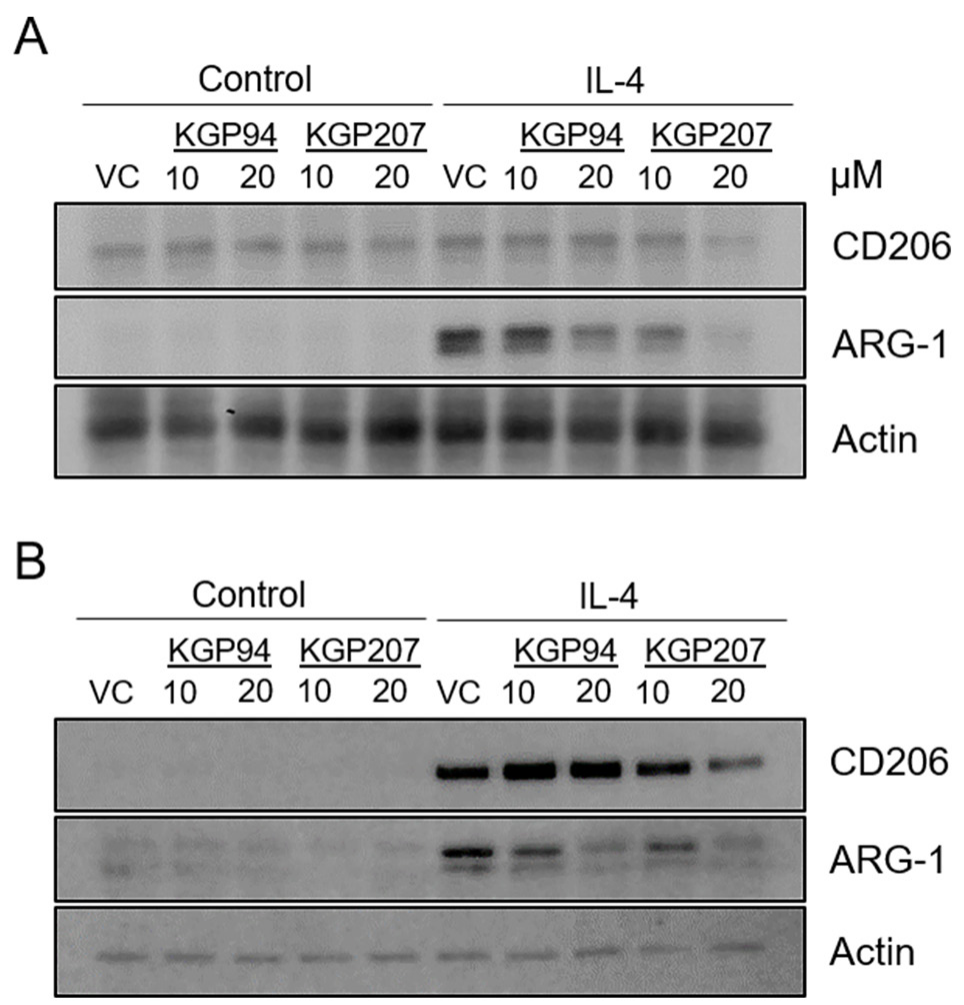
Cathepsin L inhibitors reduce expression of M2 markers **(A)** Raw264.7 and **(B)** primary bone marrow-derived macrophages were treated with 10 ng/mL IL-4 for 48 h in the presence of 10 or 20 µM KGP94 or KGP207. Whole cell lysates were analyzed by immunoblot.

Our data indicate that cathepsin L activity is important for invasion in response to IL-4, a hallmark of M2 functionality (Figure 2A). We reasoned that other aspects of M0 to M2 differentiation may also be affected by cathepsin L activity. Expression of M2 markers is another indicator of M0 to M2 differentiation [24]. Therefore, we treated Raw264.7 (Figure 5A; quantified in Supplementary Figure 2E) or primary bone marrow-derived macrophages (Figure 5B; quantified in Supplementary Figure 2F) with the cathepsin L inhibitors KGP94 and KGP207 prior to stimulation with IL-4. Immunoblot analysis revealed that treatment with KGP94 or KGP207 resulted in decreased expression of M2 markers CD206 and Arginase-1. Together, these data raise the intriguing possibility that macrophages treated with cathepsin L inhibitors are less M2-like.

## DISCUSSION

Protease secretion is involved in multiple aspects of tumor development, and contributes to the metastatic phenotype. The lysosome-associated cysteine protease, cathepsin L, is often overexpressed in breast cancer and is believed to contribute to the dissemination of breast cancer cells [3, 12, 17, 18]. Prior clinical trials investigating the anti-cancer efficacy of protease inhibitors (most notably MMP inhibitors) have been largely unsuccessful due likely to the lack of specificity and resulting normal tissue toxicity of these agents [6, 7]. However, KGP94 and KGP207 have high specificity for cathepsins L and K, suggesting that toxicity resulting from off-target inhibition might be mitigated [13–16]. These agents have been tested in murine models and their use resulted in slower tumor growth and decreased angiogenesis and metastasis [17, 18]. Target specificity and promising pre-clinical studies suggest that KGP94 and KGP207 may be advantageous in the treatment of tumors with high expression of cathepsin L.

When designing anti-tumor therapeutics, it is important to consider the role of the tumor stroma. Macrophages are a myeloid-derived immune cell population that contribute to breast cancer growth and progression. In fact, macrophage-mediated therapies are currently being investigated as an adjuvant for triple negative breast cancers [37]. Cytokines such as IL-4 and interleukin-13 activate the M2 transcriptional program in macrophages, resulting in macrophages that are primed for tissue remodeling [24]. M2 macrophages secrete a host of growth factors and proteases to aid in normal biological processes such as wound healing and mammary gland involution [20, 24]. For example, M2 macrophages secrete vascular endothelial growth factor (VEGF) to promote angiogenesis and epidermal growth factor (EGF) to stimulate wound closure. However, in the context of tumor development, these macrophage-derived growth factors can also encourage tumor progression [21, 38].

Given the versatile and important role that macrophages play in breast cancer progression and metastasis, we are investigating therapeutically targeting macrophages for novel anticancer therapy. Previous studies have shown that tumor-associated macrophages secrete proteases to aid tumor cell invasion [31–33]. Of note, cathepsin L expression is directly regulated by IL-4, the same cytokine that stimulates M0 to M2 differentiation. Activation of the IL-4 receptor results in STAT6-dependent transcriptional activation of the cathepsin L gene [33]. The increased expression of cathepsin L in response to IL-4 stimulation was similarly observed in our studies (Figure 1). Our data indicate that cathepsin L supplied from macrophages is important for tumor cell invasion *in vitro* (Figure 4). This finding fits with results of prior investigations showing that tumor-associated macrophages supply proteases to increase tumor cell invasion [31–34].

Surprisingly, we found that treatment with KGP94 and KGP207 resulted in the reduced expression of the M2 markers Arginase-1 and CD206 (Figure 5). While not definitive, these data could indicate that cathepsin L inhibition results in macrophages that are less M2-like. However, these data raise the question of how cathepsin L is regulating the expression profile of macrophage markers. Cathepsin L is primarily found within the lysosome and acts to degrade lysosomal contents as part of the normal endocytic pathway or autophagy [4]. Cathepsin L can also be secreted from cells and aids in proteolytic processing of the extracellular matrix and the activation of other proteases [4]. However, inhibition of both lysosomal and extracellular cathepsin L are not likely to cause a decrease in the expression of M2 markers. A lesser known function of cathepsin L is its role in regulating the transcriptional activator, CCAAT-displacement protein/cut homeobox (CDP/Cux) in the nucleus [39, 40]. Nuclear cathepsin L cleaves Cux, resulting in the activation of the Cux transcription factor [39]. While Cux activity is known to decrease the expression of M1 cytokines, it raises the titillating possibility that Cux also regulates the expression of M2 cytokines [41]. More detailed studies are needed to determine the mechanism by which cathepsin L inhibition modulates the expression of M2 markers.

Overall, these studies indicate that cathepsin L from both the neoplastic cell and macrophage populations contribute to tumor cell invasion. Because cathepsin L is expressed and secreted by both neoplastic and macrophage populations, its inhibition could be therapeutically beneficial for breast cancers with high macrophage infiltration.

## MATERIALS AND METHODS

### Reagents

IL-4 and macrophage colony stimulating factor (M-CSF) were purchased from R&D systems. Both IL-4 and M-CSF were used at 10 ng/mL. KGP94 and KGP207 were a generous gift from Dr. Kevin Pinney (Baylor University) and prepared in 10 mM stock solutions in DMSO.

### Immunoblot

Whole cell lysates were harvested on ice by scraping into RIPA buffer containing protease inhibitor (Sigma Aldrich). Samples were incubated on ice and vortexed intermittently over 30 minutes then centrifuged at 10,000 RPM for 10 minutes at 4°C. Protein concentration was assessed by BCA assay and equal amounts of protein were diluted into laemmli buffer (0.125M Tris, 4% SDS, 20% glycerol, β-mercaptoethanol). Samples were boiled for 5 minutes before proceeding to immunoblot. Whole cell lysates were run on polyacrylamide gels prior to transfer to PVDF membrane. Membrane was blocked for 1 hour in 5% milk TBST (20mM Tris, 137mM NaCl, 0.1% Tween 20, pH 7.5). Primary antibodies were diluted in 5% bovine serum albumin in TBST and incubated overnight at 4°C followed by detection using HRP-conjugated secondary antibodies and Pierce ECL2 (ThermoFisher Scientific, Waltham, MA, USA).

### Antibodies

Arginase-1 1:1000 (Cell Signaling Technologies, Beverly, MA, USA). Cathepsin L 1:2000 (R&D Systems, Minneapolis, MN, USA). CD206 1:1000 (ThermoFisher Scientific). Actin 1:20,000 (Sigma-Aldrich, St. Louis, MO, USA)

### Cell culture

Raw264.7 cells (ATCC, Manassas, VA, USA) were grown in 10% fetal bovine serum (FBS) in DMEM. 4T1 cells (ATCC) were cultured in 10% FBS RPMI. Non target (SHC202V) and cathepsin L knockdown (4T1: KD1-TRCN0000030579 and KD2-TRCN0000030580; Raw264.7: KD1- TRCN0000030579 and KD2-TRCN0000030583) sublines were generated using Mission Lentivirus Transduction Particles (Sigma-Aldrich, St. Louis, MO, USA) according to the manufacturer’s protocol. Sublines were cultured in the presence of 4 µg/mL puromycin. All cells were grown at 37°C and 5% CO_2_ and subcultured upon reaching 75% confluence.

### Bone marrow derived macrophages

Bone marrow was harvested from the hind limbs of female Balb/c mice [42]. Cells were plated in 10% FBS DMEM with 10 ng/mL recombinant mouse M-CSF for 48 hours. Non-adherent cells were washed off and adherent macrophages were maintained in M-CSF for the experimental duration.

### Enzyme linked immunosorbent assay (ELISA)

Raw264.7 or bone marrow derived macrophages were plated in 100 mm dishes and treated with vehicle or 10 ng/mL IL-4 for 24 hours in complete media. Cells were then washed to remove serum residue and then incubated with vehicle or 10 ng/mL IL-4 for 24 hours in serum free media. Conditioned-media was collected and concentrated using Amicon 10K concentrators (Millipore, Burlington, MA, USA). Mouse cathepsin L levels were determined using ELISA (MyBiosource, San Diego, CA, USA) and normalized to 10^6^ cells.

### Conditioned media

Cells (10^6^) were plated in 100 mm dishes and allowed to adhere for 24 hours. Media was removed and cells were washed with PBS to ensure removal of any non-adherent cells. For Boyden chamber assays, 10 mL of complete media was added. For ELISA, 10 mL of serum free media was added. Cells were incubated at 37°C for an additional 24 hours. Conditioned media was harvested, centrifuged at 1000 RPM for 10 minutes at 4°C to pellet out any cells, and then immediately used for Boyden chamber experiments or ELISA.

### Boyden chamber assays

Both motility and invasion assays were performed using 8 µm pore size Boyden chamber inserts. For invasion assays, the membrane was coated with a 1:5 dilution of Matrigel in serum free media. Matrigel-coated inserts were incubated at 37°C for 30 minutes prior to the start of the assay. Conditioned media from either 4T1 or Raw264.7 cells was collected and used as a chemoattractant on the bottom of the insert. For studies involving cathepsin L inhibition, vehicle or drug was added to both the top and bottom chambers of the insert. 4T1 (10^4^) or Raw264.7 (2X103) cells were plated in the top chamber of the Boyden inserts and allowed to invade/migrate for 24 hours. Cells were fixed and stained with isopropanol and crystal violet.

### Densitometry

Quantification of immunoblots was performed using ImageJ software. The images were inverted and the integrated density for each ban was determined using ImageJ analysis. The integrated density of the band of interest was divided by housekeeping gene band in the same lane to generate a measurement of band intensity.

### Statistics

Statistical analysis was performed using GraphPad software of at least three independent data sets. Statistical significance of a minimum p-value <0.05 was determined using Mann-Whitney *t* Test (two-tailed). Data are represented as means ± SEM.

## SUPPLEMENTARY MATERIALS FIGURES





## Abbreviations

ELISA: Enzyme linked immunosorbent assay
KD: Knockdown
STAT: Signal Transducer and Activator of Transcription
NT: Non Target
IL-4: Interleukin-4
Mφ: Macrophage.

## References

[1] Siegel RL , Miller KD , Jemal A . Cancer Statistics, 2017. CA Cancer J Clin. 2017; 67:7–30. 10.3322/caac.21387. 28055103

[2] Mason SD , Joyce JA . Proteolytic networks in cancer. Trends Cell Biol. 2011; 21:228–37. 10.1016/j.tcb.2010.12.002. 21232958 PMC3840715

[3] Koblinski JE , Ahram M , Sloane BF . Unraveling the role of proteases in cancer. Clin Chim Acta. 2000; 291:113–35. 10.1016/S0009-8981(99)00224-7. 10675719

[4] Turk V , Stoka V , Vasiljeva O , Renko M , Sun T , Turk B , Turk D . Cysteine cathepsins: from structure, function and regulation to new frontiers. Biochim Biophys Acta. 2012; 1824:68–88. 10.1016/j.bbapap.2011.10.002. 22024571 PMC7105208

[5] Page-McCaw A , Ewald AJ , Werb Z . Matrix metalloproteinases and the regulation of tissue remodelling. Nat Rev Mol Cell Biol. 2007; 8:221–33. 10.1038/nrm2125. 17318226 PMC2760082

[6] Overall CM , Kleifeld O . Tumour microenvironment - opinion: validating matrix metalloproteinases as drug targets and anti-targets for cancer therapy. Nat Rev Cancer. 2006; 6:227–39. 10.1038/nrc1821. 16498445

[7] Krüger A , Kates RE , Edwards DR . Avoiding spam in the proteolytic internet: future strategies for anti-metastatic MMP inhibition. Biochim Biophys Acta. 2010; 1803:95–102. 10.1016/j.bbamcr.2009.09.016. 19800374

[8] Jedeszko C , Sloane BF . Cysteine cathepsins in human cancer. Biol Chem. 2004; 385:1017–27. 10.1515/BC.2004.132. 15576321

[9] Berdowska I . Cysteine proteases as disease markers. Clin Chim Acta. 2004; 342:41–69. 10.1016/j.cccn.2003.12.016. 15026265

[10] Chauhan SS , Goldstein LJ , Gottesman MM . Expression of cathepsin L in human tumors. Cancer Res. 1991; 51:1478–81. https://cancerres.aacrjournals.org/content/51/5/1478.long. 1997186

[11] Foekens JA , Kos J , Peters HA , Krasovec M , Look MP , Cimerman N , Meijer-van Gelder ME , Henzen-Logmans SC , van Putten WL , Klijn JG . Prognostic significance of cathepsins B and L in primary human breast cancer. J Clin Oncol. 1998; 16:1013–21. 10.1200/JCO.1998.16.3.1013. 9508185

[12] Sudhan DR , Siemann DW . Cathepsin L targeting in cancer treatment. Pharmacol Ther. 2015; 155:105–16. 10.1016/j.pharmthera.2015.08.007. 26299995 PMC4624022

[13] Parker EN , Song J , Kishore Kumar GD , Odutola SO , Chavarria GE , Charlton-Sevcik AK , Strecker TE , Barnes AL , Sudhan DR , Wittenborn TR , Siemann DW , Horsman MR , Chaplin DJ , et al. Synthesis and biochemical evaluation of benzoylbenzophenone thiosemicarbazone analogues as potent and selective inhibitors of cathepsin L. Bioorg Med Chem. 2015; 23:6974–92. 10.1016/j.bmc.2015.09.036. 26462052 PMC4824049

[14] Chavarria GE , Horsman MR , Arispe WM , Kumar GD , Chen SE , Strecker TE , Parker EN , Chaplin DJ , Pinney KG , Trawick ML . Initial evaluation of the antitumour activity of KGP94, a functionalized benzophenone thiosemicarbazone inhibitor of cathepsin L. Eur J Med Chem. 2012; 58:568–72. 10.1016/j.ejmech.2012.10.039. 23168380

[15] Kumar GD , Chavarria GE , Charlton-Sevcik AK , Yoo GK , Song J , Strecker TE , Siim BG , Chaplin DJ , Trawick ML , Pinney KG . Functionalized benzophenone, thiophene, pyridine, and fluorene thiosemicarbazone derivatives as inhibitors of cathepsin L. Bioorg Med Chem Lett. 2010; 20:6610–15. 10.1016/j.bmcl.2010.09.026. 20933415

[16] Song J , Jones LM , Kumar GD , Conner ES , Bayeh L , Chavarria GE , Charlton-Sevcik AK , Chen SE , Chaplin DJ , Trawick ML , Pinney KG . Synthesis and biochemical evaluation of thiochromanone thiosemicarbazone analogues as inhibitors of cathepsin L. ACS Med Chem Lett. 2012; 3:450–53. 10.1021/ml200299g. 24900494 PMC4025852

[17] Sudhan DR , Siemann DW . Cathepsin L inhibition by the small molecule KGP94 suppresses tumor microenvironment enhanced metastasis associated cell functions of prostate and breast cancer cells. Clin Exp Metastasis. 2013; 30:891–902. 10.1007/s10585-013-9590-9. 23748470 PMC3823630

[18] Sudhan DR , Rabaglino MB , Wood CE , Siemann DW . Cathepsin L in tumor angiogenesis and its therapeutic intervention by the small molecule inhibitor KGP94. Clin Exp Metastasis. 2016; 33:461–73. 10.1007/s10585-016-9790-1. 27055649 PMC5378387

[19] Campbell MJ , Tonlaar NY , Garwood ER , Huo D , Moore DH , Khramtsov AI , Au A , Baehner F , Chen Y , Malaka DO , Lin A , Adeyanju OO , Li S , et al. Proliferating macrophages associated with high grade, hormone receptor negative breast cancer and poor clinical outcome. Breast Cancer Res Treat. 2011; 128:703–11. 10.1007/s10549-010-1154-y. 20842526 PMC4657137

[20] O’Brien J , Martinson H , Durand-Rougely C , Schedin P . Macrophages are crucial for epithelial cell death and adipocyte repopulation during mammary gland involution. Development. 2012; 139:269–75. 10.1242/dev.071696. 22129827 PMC13621425

[21] Goswami S , Sahai E , Wyckoff JB , Cammer M , Cox D , Pixley FJ , Stanley ER , Segall JE , Condeelis JS . Macrophages promote the invasion of breast carcinoma cells via a colony-stimulating factor-1/epidermal growth factor paracrine loop. Cancer Res. 2005; 65:5278–83. 10.1158/0008-5472.CAN-04-1853. 15958574

[22] Cho HJ , Jung JI , Lim DY , Kwon GT , Her S , Park JH , Park JH . Bone marrow-derived, alternatively activated macrophages enhance solid tumor growth and lung metastasis of mammary carcinoma cells in a Balb/C mouse orthotopic model. Breast Cancer Res. 2012; 14:R81. 10.1186/bcr3195. 22616919 PMC3446344

[23] Qian B , Deng Y , Im JH , Muschel RJ , Zou Y , Li J , Lang RA , Pollard JW . A distinct macrophage population mediates metastatic breast cancer cell extravasation, establishment and growth. PLoS One. 2009; 4:e6562. 10.1371/journal.pone.0006562. 19668347 PMC2721818

[24] Italiani P , Boraschi D . From Monocytes to M1/M2 Macrophages: phenotypical vs. Functional Differentiation. Front Immunol. 2014; 5:514. 10.3389/fimmu.2014.00514. 25368618 PMC4201108

[25] Mantovani A , Sozzani S , Locati M , Allavena P , Sica A . Macrophage polarization: tumor-associated macrophages as a paradigm for polarized M2 mononuclear phagocytes. Trends Immunol. 2002; 23:549–55. 10.1016/S1471-4906(02)02302-5. 12401408

[26] Wang HW , Joyce JA . Alternative activation of tumor-associated macrophages by IL-4: priming for protumoral functions. Cell Cycle. 2010; 9:4824–35. 10.4161/cc.9.24.14322. 21150330 PMC3047808

[27] Lamagna C , Aurrand-Lions M , Imhof BA . Dual role of macrophages in tumor growth and angiogenesis. J Leukoc Biol. 2006; 80:705–13. 10.1189/jlb.1105656. 16864600

[28] Williams CB , Yeh ES , Soloff AC . Tumor-associated macrophages: unwitting accomplices in breast cancer malignancy. NPJ Breast Cancer. 2016; 2:15025. 10.1038/npjbcancer.2015.25. 26998515 PMC4794275

[29] Pollard JW . Tumour-educated macrophages promote tumour progression and metastasis. Nat Rev Cancer. 2004; 4:71–78. 10.1038/nrc1256. 14708027

[30] Poh AR , Ernst M . Targeting Macrophages in Cancer: From Bench to Bedside. Front Oncol. 2018; 8:49. 10.3389/fonc.2018.00049. 29594035 PMC5858529

[31] Reddy VY , Zhang QY , Weiss SJ . Pericellular mobilization of the tissue-destructive cysteine proteinases, cathepsins B, L, and S, by human monocyte-derived macrophages. Proc Natl Acad Sci USA. 1995; 92:3849–53. 10.1073/pnas.92.9.3849. 7731994 PMC42059

[32] Gocheva V , Wang HW , Gadea BB , Shree T , Hunter KE , Garfall AL , Berman T , Joyce JA . IL-4 induces cathepsin protease activity in tumor-associated macrophages to promote cancer growth and invasion. Genes Dev. 2010; 24:241–55. 10.1101/gad.1874010. 20080943 PMC2811826

[33] Brignull LM , Czimmerer Z , Saidi H , Daniel B , Villela I , Bartlett NW , Johnston SL , Meira LB , Nagy L , Nohturfft A . Reprogramming of lysosomal gene expression by interleukin-4 and Stat6. BMC Genomics. 2013; 14:853. 10.1186/1471-2164-14-853. 24314139 PMC3880092

[34] Balce DR , Li B , Allan ER , Rybicka JM , Krohn RM , Yates RM . Alternative activation of macrophages by IL-4 enhances the proteolytic capacity of their phagosomes through synergistic mechanisms. Blood. 2011; 118:4199–208. 10.1182/blood-2011-01-328906. 21846901

[35] Olson OC , Joyce JA . Cysteine cathepsin proteases: regulators of cancer progression and therapeutic response. Nat Rev Cancer. 2015; 15:712–29. 10.1038/nrc4027. 26597527

[36] Sudhan DR , Pampo C , Rice L , Siemann DW . Cathepsin L inactivation leads to multimodal inhibition of prostate cancer cell dissemination in a preclinical bone metastasis model. Int J Cancer. 2016; 138:2665–77. 10.1002/ijc.29992. 26757413 PMC4805507

[37] Niu M , Valdes S , Naguib YW , Hursting SD , Cui Z . Tumor-Associated Macrophage-Mediated Targeted Therapy of Triple-Negative Breast Cancer. Mol Pharm. 2016; 13:1833–42. 10.1021/acs.molpharmaceut.5b00987. 27074028 PMC4899190

[38] Barbera-Guillem E , Nyhus JK , Wolford CC , Friece CR , Sampsel JW . Vascular endothelial growth factor secretion by tumor-infiltrating macrophages essentially supports tumor angiogenesis, and IgG immune complexes potentiate the process. Cancer Res. 2002; 62:7042–49. https://cancerres.aacrjournals.org/content/62/23/7042.long. 12460925

[39] Burton LJ , Dougan J , Jones J , Smith BN , Randle D , Henderson V , Odero-Marah VA . Targeting the Nuclear Cathepsin L CCAAT Displacement Protein/Cut Homeobox Transcription Factor-Epithelial Mesenchymal Transition Pathway in Prostate and Breast Cancer Cells with the Z-FY-CHO Inhibitor. Mol Cell Biol. 2017; 37:e00297–16. 10.1128/MCB.00297-16. 27956696 PMC5311241

[40] Burton LJ , Henderson V , Liburd L , Odero-Marah VA . Snail transcription factor NLS and importin β1 regulate the subcellular localization of Cathepsin L and Cux1. Biochem Biophys Res Commun. 2017; 491:59–64. 10.1016/j.bbrc.2017.07.039. 28698143 PMC5568889

[41] Kühnemuth B , Mühlberg L , Schipper M , Griesmann H , Neesse A , Milosevic N , Wissniowski T , Buchholz M , Gress TM , Michl P . CUX1 modulates polarization of tumor-associated macrophages by antagonizing NF-κB signaling. Oncogene. 2015; 34:177–87. 10.1038/onc.2013.530. 24336331

[42] Zhang X , Goncalves R , Mosser DM . The isolation and characterization of murine macrophages. Curr Protoc Immunol. 2008; 83:14.1.1–14.1.14. 10.1002/0471142735.im1401s83. 19016445 PMC2834554

