# Supplementary material for: Cathepsin L secretion by host and neoplastic cells potentiates invasion
Source: Oncotarget. 2019 Sep 17;10(53):5560–8. doi: 10.18632/oncotarget.27182 (PMC6756864; doi:10.18632/oncotarget.27182)
Supplement: Supplementary file 1 [file oncotarget-10-5560-s001.pdf]

Cathepsin L secretion by host and neoplastic cells potentiates invasion

SUPPMENTARY MATERIALS FIGURE

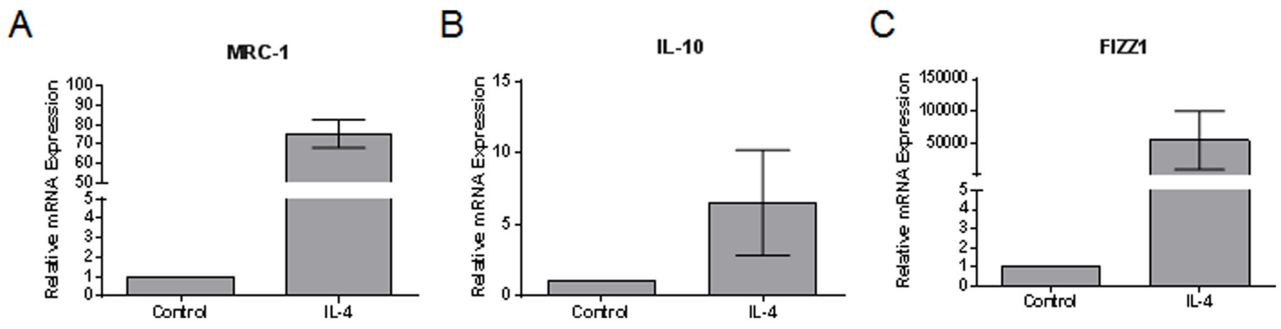

Supplementary Figure 1: IL-4 upregulates the expression of M2-associated gene products. Raw264.7 macrophages were stimulated with 10 ng/mL IL-4 for 48 h. Relative mRNA expression for **(A)** MRC-1, **(B)** IL-10, and **(C)** FIZZ1 was analyzed by semi-quantitative RT-PCR.
