# Supplementary material for: Cathepsin L secretion by host and neoplastic cells potentiates invasion
Source: Oncotarget. 2019 Sep 17;10(53):5560–8. doi: 10.18632/oncotarget.27182 (PMC6756864; doi:10.18632/oncotarget.27182)
Supplement: Supplementary file 2 [file oncotarget-10-5560-s002.pdf]

Cathepsin L secretion by host and neoplastic cells potentiates invasion

SUPPMENTARY MATERIALS FIGURE

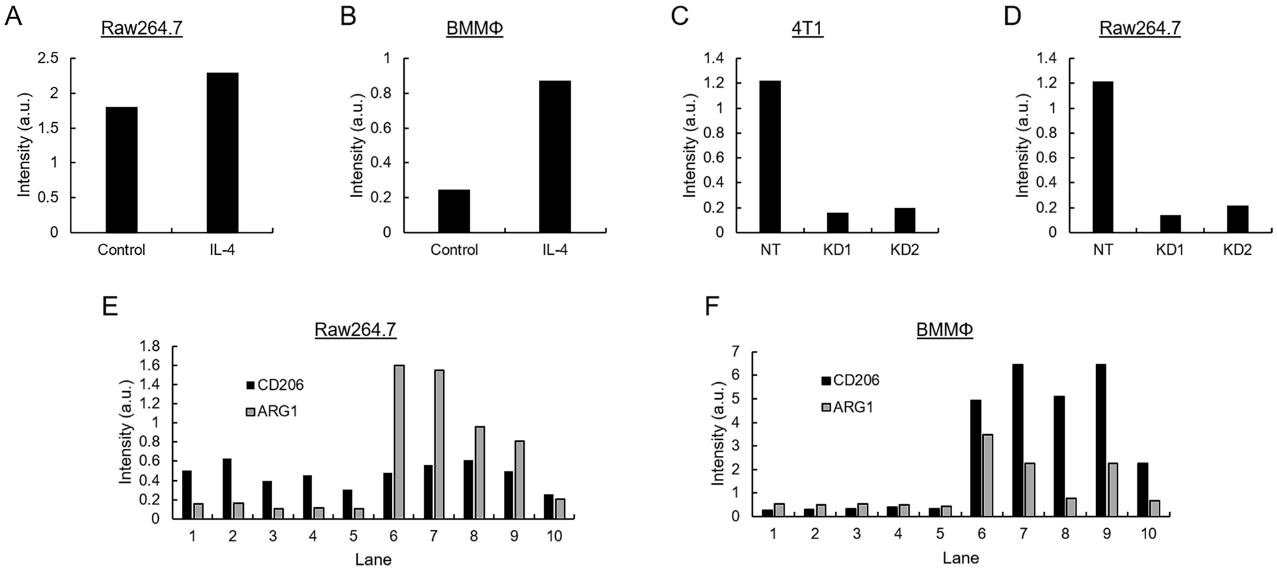

Supplementary Figure 2: Densitometry quantification of immunoblots. (A) Densitometry for Figure 1A. **(B)** Densitometry for Figure 1C. **(C)** Densitometry for Figure 4A. **(D)** Densitometry for Figure 4D. **(E)** Densitometry for Figure 5A. **(F)** Densitometry for Figure 5B.
